# Artificial Respiration in Chloroform Collapse

**Published:** 1894-05-12

**Authors:** Benjamin Ward Richardson


### Artificial Respiration in Chloroform Collapse.

By Sir Benjamin Ward Richardson, M.D., F.R.S.

In addition to artificial respiration other methods for exciting respiration and circulation have been suggested, such as the application of ammonia to the nostrils, and the process of fillipping the body with a towel. In experiment I never witnessed any good effects from any method of this kind, but I have twice produced temporary good results by setting up artificial respiration with air slightly charged with ammonia vapour. I also saw in the lower animals a remarkable recovery from uniting ammonia vapour with pure oxygen for artificial respiration, and it is certainly worthy of trial in the human subject that ammonia should be so combined and used. This could be done by letting the air supplied for the respiratory purpose pass through a half-pint Wolff's bottle containing a few minims of liquid ammonia within the bottle. The effect of ammoniacal air and also of ammoniated oxygen is to excite several very quick respirations, which continue after the ammonia vapour is withdrawn, and which, in one instance, passed into natural respiration with recovery, oxygen being the diluting medium.

### Inversion of the Body as an Adjunct to Artificial Respiration.

Various other modes of treating the collapsed under chloroform have been suggested, and one especially deserves careful consideration because it goes well with artificial respiration, and is not difficult to carry out. This consists in inverting the body of the patient, letting the body be suspended head downwards, so as to allow the blood to find its way from the right side of the heart into the lungs by gravitation. So far back as 1853-4 I illustrated this method by experiments on animals recently dead from chloroform. I showed that when an animal had quite ceased to breathe, and all signs of life were destroyed, if the thorax were laid open and the heart were found making feeble contractions, the motion of both the auricles and the ventricles could be invigorated by holding the animal with the head straight downwards. In that position the blood in the inferior cava made its way into the right auricle, filled the ventricle, and caused a much more determined contraction, which was greatly improved by continuing artificial respiration at the same time. Some years later M. Nelaton in a case of chloroform collapse inverted the body of the patient with the most remarkable results. Faint breathing recommenced, and ultimately recovery from what appeared to be absolute death. The recovery here seemed to occur without the aid of artificial respiration. Two years ago Dr. A. E. Prince, of Illinois, employed a similar practice to that of Nelaton but with the addition of artificial respiration, and with a result equally striking. Dr. Prince suspended the body of a collapsed patient, by bringing the legs over the shoulders of his assistant, who then moved about, carrying apparently a lifeless body, while Dr. Prince inflated the lungs by direct inflation. The result was most satisfactory. The diaphragm was raised by the artificial filling of the thorax, and when the tension was removed the chest by the descent of the diaphragm and by the pressure of the viscera resting upon it was well emptied; in time the natural respiration recommenced, and the patient was restored to life. This was a brilliant success, but it must not raise too great expectations. It was an instance in which the column of blood from the right to the left side of the heart, a column not completely broken, was sufficiently quickened by the movement to allow of recovery. In symptoms of collapse from chloroform in the first stage, where syncopal apnœa is the mode of death, this method is most promising, and it strongly suggests the introduction of an operation table into our hospitals; a table which would admit of an up and down movement, so that the head of the patient could be brought down and the body inverted at a moment's notice.

### Injection of the Veins.

I noticed in my last paper the three degrees of motion of the heart, and indicated that in the third or low degree of motion the auricular and ventricular action may be present, but with no sufficient propulsive power to carry the blood over from the right to the left side of the heart. It might, therefore, be supposed that by forcing a current of blood or of a saline fluid into the veins, as in transfusion, the heart might be spurred into vigorous action, and that the circulation could so be restored. I have put this to the test in various ways, especially by the process of transfusing blood held fluid by ammonia, and warmed up to the natural degree. The phenomena produced were remarkable. Under this stimulation the heart made distinct beats, which could be felt through the thorax, but with a quick cessation of activity. The explanation of the cessation is as important as it is interesting. I found that for a column of blood to be maintained through the body, both on the venous and the arterial side, the column must always be continuous. It may be very small in size, but it must be in one unbroken line. The circulation, in fact, is carried on on the syphon principle; that is to say, in life the blood is in motion exactly like a column of fluid passing through a syphon. So long as there is a continuous column of blood, arterial, capillary, and venous, there is a possibility of recovery; but, once let the column be broken in any part of its course, and no force resident in the circulation will restore life by any method as yet discovered.

I have tried various plans for rejoining the broken column; (*a*) by extreme exhaustion of the lungs through the trachea with sudden admission of air; (*b*) by tying a tube in the abdominal aorta and endeavouring by an exhaust pump to draw over the blood from the right to the left side of the heart; (*c*) by setting up artificial respiration from the abdominal cavity, with the body inverted; (*d*) by a series of rapid changes in the position of the body, from the inverted to the upright position. Up to this time there has been no affirmative success from these methods.

One experimental result in this direction must, however, be recorded, because of the singular and hopeful fact that followed. I thought it possible that by injecting into the peritoneum of an animal recently dead from chloroform, a ten-volume solution of peroxide of hydrogen, oxygen would be liberated in the peritoneal cavity, would be absorbed by the large veins, and by what may be called a *vis a tergo*, would force a column of blood through the right side of the heart, over the surface of the lungs. The effect was most peculiar; respiration was re-established, the movements of the heart could be felt through the thorax, and recovery seemed pronounced. Then there was a sudden arrest of every sign of life, and when death was unmistakable, the post-mortem examination told the story. The blood throughout the veins was florid, the auricle and ventricle of the heart were tense with blood, the pulmonary artery was filled, the lungs were filled, but the blood was charged with bubbles of oxygen gas, which, acting after the manner of air blown into a vein, caused mechanical obstruction to the course of the blood, and by this means prevented the re-establishment of the current.

In summary, at the present moment, I can leave but one practical lesson on this great subject, namely; that in death from chloroform, gentle artificial respiration by simple inflation of the lungs through the nostrils, with the body inverted, is the only rational means of resuscitation.

